# Characterization and productivity profiles of *Aedes aegypti* (L.) breeding habitats across rural and urban landscapes in western and coastal Kenya

**DOI:** 10.1186/s13071-017-2271-9

**Published:** 2017-07-12

**Authors:** Harun N. Ngugi, Francis M. Mutuku, Bryson A. Ndenga, Peter S. Musunzaji, Joel O. Mbakaya, Peter Aswani, Lucy W. Irungu, Dunstan Mukoko, John Vulule, Uriel Kitron, Angelle D. LaBeaud

**Affiliations:** 1 Department of Biological Sciences, Chuka University, Chuka, Kenya; 2 Department of Environment and Health Sciences, Technical University of Mombasa, Mombasa, Kenya; 3 Centre for Global Health Research, Kenya Medical Research Institute, Kisumu, Kenya; 4 Vector Borne Disease Unit, Msambweni, Kenya; 5 Department of Zoology, School of Biological Sciences University of Nairobi, Nairobi, Kenya; 6 Vector Borne Disease Unit, Center for Global Health and Diseases, Nairobi, Kenya; 7 Department of Environmental Sciences, Emory University, Atlanta, GA USA; 8 Department of Pediatrics, Division of Infectious Diseases, Stanford University School of Medicine, Stanford, California, USA

**Keywords:** *Aedes aegypti*, Larval habitats, Productivity, Western and coastal Kenya, Household breeding surveys

## Abstract

**Background:**

*Aedes aegypti*, the principal vector for dengue and other emerging arboviruses, breeds preferentially in various man-made and natural container habitats. In the absence of vaccine, epidemiological surveillance and vector control remain the best practices for preventing dengue outbreaks. Effective vector control depends on a good understanding of larval and adult vector ecology of which little is known in Kenya. In the current study, we sought to characterize breeding habitats and establish container productivity profiles of *Ae*. *aegypti* in rural and urban sites in western and coastal Kenya.

**Methods:**

Twenty sentinel houses in each of four study sites (in western and coastal Kenya) were assessed for immature mosquito infestation once a month for a period of 24 months (June 2014 to May 2016). All water-holding containers in and around the households were inspected for *Ae*. *aegypti* larvae and pupae.

**Results:**

Collections were made from a total of 22,144 container visits: Chulaimbo (7575) and Kisumu (8003) in the west, and from Msambweni (3199) and Ukunda (3367) on the coast. Of these, only 4–5.6% were positive for *Ae*. *aegypti* immatures. In all four sites, significantly more positive containers were located outdoors than indoors. A total of 17,537 *Ae*. *aegypti* immatures were sampled from 10 container types. The most important habitat types were buckets, drums, tires, and pots, which produced over 75% of all the pupae. Key outdoor containers in the coast were buckets, drums and tires, which accounted for 82% of the pupae, while pots and tires were the only key containers in the western region producing 70% of the pupae. Drums, buckets and pots were the key indoor containers, producing nearly all of the pupae in the coastal sites. No pupae were collected indoors in the western region. The coastal region produced significantly more *Ae*. *aegypti* immatures than the western region both inside and outside the sentinel houses.

**Conclusions:**

These results indicate that productive *Ae*. *aegypti* larval habitats are abundant outdoors and that only a few containers produce a majority of the pupae. Although the numbers were lower, productive habitats were detected within households. Targeting source reduction efforts towards these productive containers both inside and outside homes is likely to be a cost-effective way to reduce arboviral transmission in these regions.

## Background

*Aedes aegypti*, the principal vector for dengue, chikungunya, and other emerging arboviruses, is closely associated with human dwellings in endemic areas, and breeds preferentially in natural and man-made container habitats of various characteristics [1–5]. In Kenya, the first dengue outbreak was reported in 1982 [6], with more recent outbreaks reported in Mombasa [7] and the northeastern town of Mandera [8]. These recent dengue outbreaks involved three different dengue virus (DENV) serotypes (DENV-1, DENV-2 and DENV-3) [7, 8]. In Kenya, dengue appears to be endemic with periodic outbreaks primarily occurring along the coast in recent years [7–9]. Earlier studies reported seroprevalence of 1.1% to 7.5% in western Kenya outside the known epidemic periods [10, 11]. Similar studies in coastal Kenya revealed DENV seroprevalence ranging from 1% to 53% [11, 12].

In Kenya, the two subspecific taxa, *Ae*. *aegypti aegypti* and *Ae*. *aegypti formosus* are the primary dengue vectors [13] with the latter being more prevalent in western Kenya [14]. *Aedes ae*. *aegypti* is more common in human dwellings, while *Ae*. *ae*. *formosus*, the sylvatic form, breeds in forest treeholes [13, 15]. The two subspecies were found to occur sympatrically along the coast of Kenya [13]. Larvae of *Ae*. *aegypti* and *Culex* spp. are often reported co-occurring in the same breeding sites [16–18]. In the coastal town of Malindi, *Ae*. *aegypti* immatures were found both indoors and outdoors at high numbers [19]. In a recent study [20], *Ae*. *aegypti* was reported to breed mostly outdoors, in what may be a novel adaptive strategy by this vector, which traditionally is considered to have adapted to the domestic environment and breed mainly in indoor water storage containers [13, 21, 22]. Predominantly outdoor breeding by *Ae*. *aegypti* has also been observed in the city of Mombasa, where recent outbreaks of dengue have been reported [23]. The outdoor environment offers more breeding opportunities due to the availability of numerous rain-filled discarded containers. Heterogeneous populations of *Ae*. *aegypti* were found to occur in such habitats for relatively long periods of time [20]. Human activities involving water storage, use and disposal of water-holding containers greatly influence *Ae*.*aegypti* breeding in individual households. Some of the key factors that may influence productivity of *Ae*.*aegypti* in different container types include, the frequency of water replenishment, the availability of food for the larvae [24], the degree of sunlight exposure [25] and container covering [26].

There are few studies on the larval habitat productivity of *Ae*. *aegypti* in Kenya [13, 14, 19], despite the evidence of dengue and chikungunya virus transmission in Kenya in recent years [7–9, 27, 28]. The presence of DENV and other arboviruses in Kenya [29], coupled with ideal ecological conditions for the vector mosquitoes, increases the risk for DENV outbreaks in this region. In the absence of a vaccine, epidemiological surveillance and vector control remain the best practices for preventing dengue outbreaks [22, 30–32]. Effective vector control depends on a good understanding of larval and adult vector ecology of which little is known in Kenya. The purpose of this study is to characterize breeding habitats and establish container productivity profiles of *Ae*.*aegypti* in rural and urban sites in western and coastal Kenya. By determining the container habitat characteristics that influence the selection of a breeding habitat by *Ae*.*aegypti* mosquitoes, key container habitats can be identified and targeted for effective larval source reduction efforts in dengue vector control programs.

## Methods

### Study sites

Mosquito surveys were conducted in the western and coastal regions of Kenya. In the coastal region two sites, each covering 25 km^2^, both in Kwale County, South Coast were studied: Ukunda, an urban site (4°17′59.9994″S, 39°34′59.8794″E), and Msambweni, a rural site (4°28′0.0114″S, 39°28′0.12″E), located approximately 30 and 60 km south of the port city of Mombasa, respectively. The coastal climate is tropical: hot and humid throughout the year with annual mean temperatures of 23–34 °C and average relative humidity of 60–80%. Ukunda is a rapidly growing urban center with population density of about 2000 people/km^2^. The area is characterized by a proliferation of unplanned residential houses, with unreliable water, sewer and waste management systems. Most residents engage in small-scale trade, fishing and casual labour in the tourist industry along the Indian Ocean coast. Msambweni is a rural area with a population density of about 460 people/km^2^, where most of the residents are fishermen and subsistence farmers. Residents rely mainly on wells and rainfall for their water for domestic use, since the piped water system is inadequate and unreliable.

In the west, two sites, each covering approximately 25 km^2^, both in Kisumu County were studied: Kisumu, an urban site located on the shore of Lake Victoria (0.1000°S, 34.7500°E elevation 1100 m), and Chulaimbo, a rural site 19 km west of Kisumu City (0.03572°S, 34.621°E, elevation 1328 m). The region has a mean annual temperature range of 12–35 °C, with an average annual rainfall of 1352 mm and average relative humidity range of 66–83%. Kisumu site has a density of about 15,000 people/km^2^, most of them in the low income bracket. About 75% of the study site falls within an unplanned settlement area and 25% within a well-planned residential area. The site has intermittent water supply system, and limited sewer and waste disposal, especially in the unplanned area. Chulaimbo has a population density of about 500 people/km^2^. Most residents are small-scale subsistence farmers.

Residents in the four study sites store water for domestic use in diverse containers because the water supply system is unreliable. Water supply in the rural study sites is mainly from harvested rainwater, wells and boreholes, and these also supplement the irregular piped water supply system characteristic of our urban study sites. The study regions are characterized by four seasons: long dry (January-March), long rainy (April-June), short dry season (July-September), and short rainy (October-December) [33–35].

### Mosquito surveys

Prior to the mosquito surveys, mapping and a demographic survey were conducted in all households in all study sites. Forty sentinel houses in each of the four study sites were then selected from the mapped houses by simple random sampling; 20 houses for larval sampling and the other 20 for ovitrap surveys. Practicability of repeated mosquito surveys in sentinel houses in each site over an extended period (2 years) guided the choice of 40 houses. During mosquito surveys the selected 20 sentinel houses were assessed for immature mosquito infestation once a month for a period of 24 months (June 2014 to May 2016). For the mosquito surveys, a household was defined as a single residential building, and its surrounding area within approximately a 10 m radius. The distance between selected households ranged from 100 to 200 m. Informed consent was obtained from all heads of households, and when a house was inaccessible, it was replaced by the nearest house.

All natural and artificial water-holding containers in and around each household were inspected for mosquito larvae and pupae. All pupae and larvae (3rd and 4th instars) from positive containers were collected with the aid of pipettes and ladles [36], counted and recorded on field-data forms. Water from large containers was first sieved and mosquito samples were placed in a white plastic tray with some water from which the immatures were pipetted. Mosquito samples were placed in 10 ml falcon tubes and/or Whirl-pak® plastic bags (Nasco, Fort Atkinson, WI), labeled, and taken to the Vector Borne Disease Control Unit (VBDCU) laboratory in Msambweni Hospital for the coastal sites or the Kenya Medical Research Institute (KEMRI) Laboratory in Kisumu for the western study sites. Immature mosquitoes were reared in 200 ml plastic cups under laboratory conditions at an average temperature of 28.15 ± 1.8 °C and relative humidity of 80.9 ± 6.3%, and larvae were fed on TetraMinbaby® fish food (Tetra Werke, Melle, Germany). Emerged adults were identified to species using standard taxonomic keys [37]. We distinguished *Ae*. *aegypti* (L.) subspecies morphologically following keys as provided by Edwards [37], Mattingly [38] and Huang [39].

### Ovitrap survey

Two modified ovitraps were placed in 20 households that were randomly selected as fixed sampling points in each of our four study sites. Each ovitrap consisted of a black plastic cup and filled with about 350 ml of tap, borehole or rainwater. The inside of the cup was lined by a brown paper towel that was partially submerged. Eggs were laid on the damp paper towel just above the water line. The indoor trap was placed on ground level in a dark sheltered location of the living or bed room. Outdoor ovitraps were placed within a 10 m radius in suitably sheltered locations. Ovitraps were set once a week every month for a period of 24 months (June 2014 to May 2016). The ovitraps were serviced [40] after 5 days. First the paper towel in each trap was removed, wrapped in white tissue paper and placed in a plastic Ziploc bag that was labeled with house number and location of the trap. Each paper towel was examined under the dissecting microscope (×40) (Nikon®, SMZ Japan) for identification and counting of *Ae*. *aegypti* eggs. To confirm the species, the eggs were submerged in seasoned tap water for hatching and larvae reared to adults which were identified using a standard taxonomic key [37].

### Classification and characterization of containers

A total of ten container types were identified and classified based on their use and material: drums, animal feeding containers (AFC), tires, pots, small domestic containers (SDC), treeholes, wells, buckets, jerrycans and others. Drums were defined as 100–500 l capacity plastic or metal water storage containers. Animal feeding containers, ranged from small 1 l bird watering and feeding containers made of plastic or cut tires, to large 300 l concrete cattle watering containers. Pots included flower vases and water storage vessels made of clay. Small domestic containers included small plastic food containers, tins, bottles, plates, cans, cooking pots (sufuria) and jars. Others included; polythene bags, fallen leaves, coconut shells, hoof prints, drains, gutters, septic tanks, shoes, cisterns and sinks. Wells were open dugout pits used for water provision in homesteads. For each breeding habitat, records were made on the container type (small domestic container, bucket, jerrycan, tire, drums, animal feeding trough, pot, tree hole, well and other) and location in the household (indoor or outdoor).

### Weather data

Daily temperature and relative humidity data was collected using HOBO data loggers (HOBO, Onset Computer Corporation, Bourne, MA, USA). Rainfall data were collected daily using event data logger rain gauges (HOBO, Onset Computer Corporation, Bourne, MA, USA) that were placed in each of the four study sites.

### Data analysis

Positive containers were those with one or more *Ae*. *aegypti* larvae or pupae. Proportion of wet containers that were positive in each site was determined and a Chi- square test was used to compare the distribution of positive containers between indoor and outdoor locations. Key larval habitats were defined as containers that are most productive for *Ae aegypti* pupae. Kruskal-Wallis test was used to compare the distribution of *Ae*. *aegypti* infestation in the four study sites, and productivity between seasons. Mosquito indices were calculated as follows: container index (the percentage of containers infested with *Ae*. *aegypti* immatures) and pupae per person index (the number of pupae over the total number of persons in a household). Data analysis was performed using SAS 9.1 statistical software (SAS Institute, Gary, NC).

## Results

### Seasonality and climate factors at four study sites

**Fig. 1:**
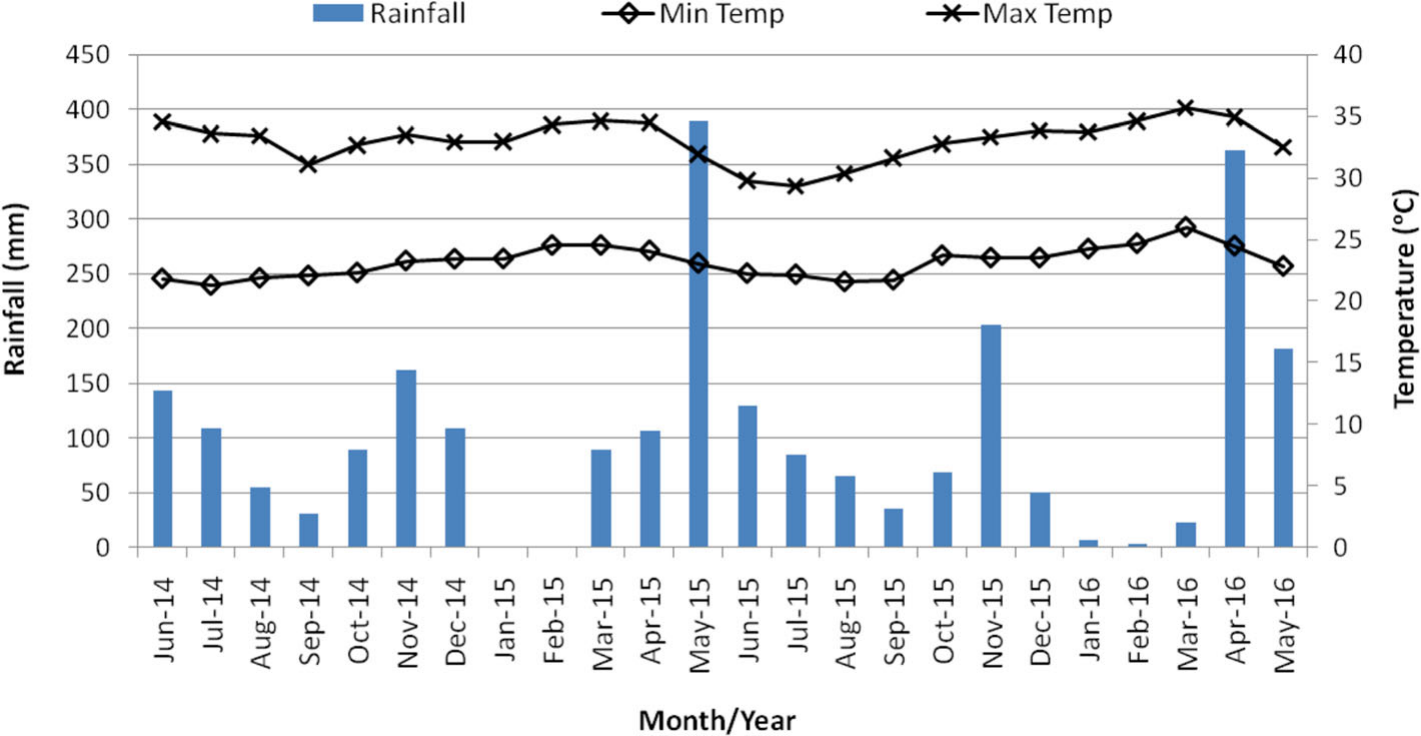
Monthly distribution of rainfall, minimum and maximum temperatures for coastal region sites from June 2014 to May 2016. Seasons: long rainy (April-June), short dry (July-September), short rainy (October-December); long dry (January-March)

**Fig. 2:**
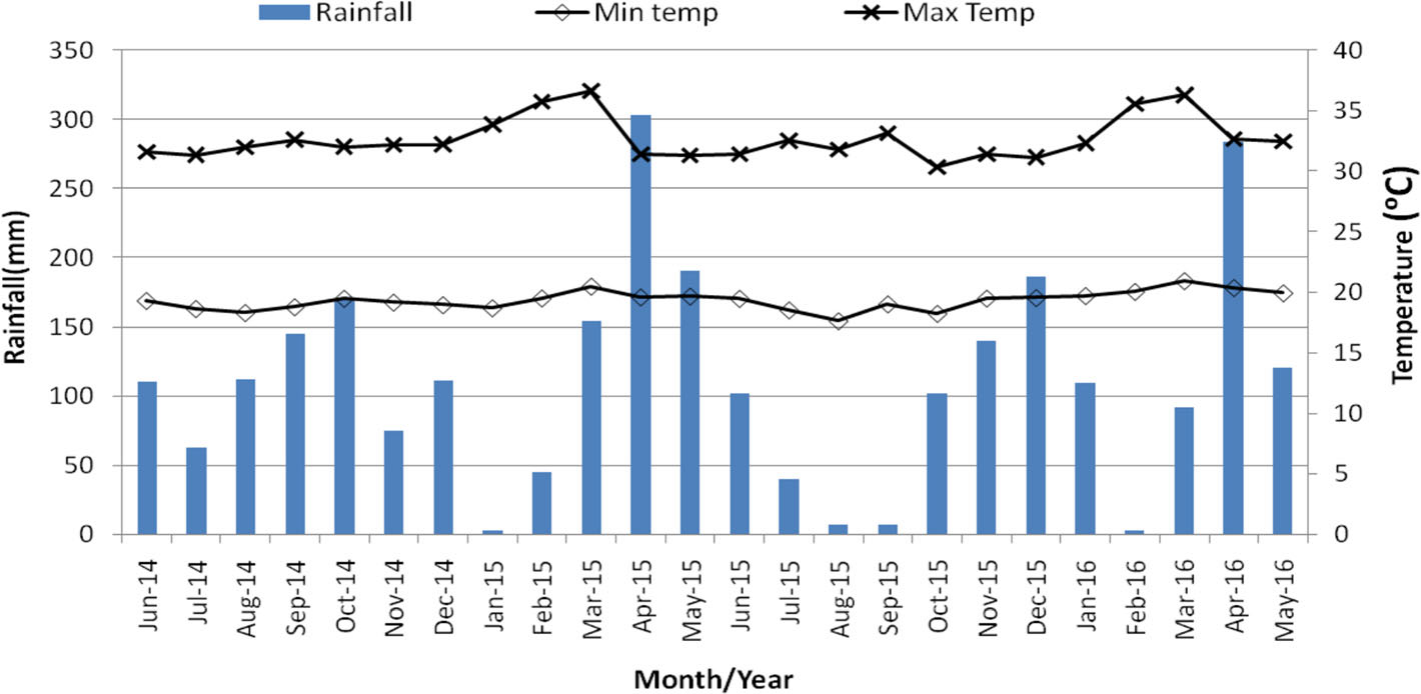
Monthly distribution of rainfall, minimum and maximum temperatures for western region sites from June 2014 to May 2016. Seasons: long rainy (April-June), short dry (July-September), short rainy (October-December); long dry (January-March)

During the study period, a mean annual temperature of 23–33 °C and annual rainfall of 1244 mm were recorded for the coast region (Fig. 1). Seasonal variations in rainfall and temperature recorded for the two coastal study sites were similar (Ukunda: mean annual temperature of 23–34 °C; average annual rainfall of 1188 mm/year and Msambweni: mean annual temperature 23–32 °C and average rainfall, 1300 mm/year). In the western region annual temperature range was 19–33 °C and average annual rainfall of 1335 mm (Fig. 2) (Kisumu: mean annual temperature 20–33 °C and rainfall 1130 mm/year and Chulaimbo: mean annual temperature 18–32 °C and rainfall 1538 mm/year).

### Frequency and presence of *Ae. aegypti* immatures in containers

Collections were made from 22,144 wet container visits: Chulaimbo (7575), Kisumu (8003), Msambweni (3199) and Ukunda (3367). During the visits, some of the containers were repeatedly sampled. Of these, only 5.49, 4.60, 5.56 and 4.54%, respectively, were positive for *Ae*. *aegypti* immatures. In all four sites, more positive containers were located outdoors (94.8%) than indoors (5.2%) (*χ* ^2^ = 895.1, *df* = 1, *P* < 0.0001; Tables 1, 2). There was a significant variation in the proportion of *Ae*. *aegypti* positive containers in outdoor locations among the four sites (*χ* ^2^ = 8.98, *df* = 3, *P* < 0.05 (Chulaimbo: 38.5%; Kisumu: 33.9%; Msambweni: 15.5%; and Ukunda: 12.1%). Despite the low presence of *Ae*. *aegypti-*positive containers indoors, the proportion of wet containers indoors was higher (52.8%) than that outdoors (47.2%) in all the study sites (*χ* ^2^ = 67.7, *df* = 1, *P* < 0.0001). However, for the indoor container habitats, a much higher proportion of *Ae*. *aegypti*-positive containers was observed in the coastal site of Ukunda (43.1%) than in the other sites (Chulaimbo 12.1%, Kisumu, 13.8%, Msambweni 31%) (*χ* ^2^ = 3, *P* < 0.0001; Table 2).

**Table 1:** Containers with larvae and pupae for outdoor locations for western [Chulaimbo: Rural (R) and Kisumu: Urban (U)] sites and coastal [Msambweni (R) and Ukunda (U)] sites, Kenya

Container type	Western						Coastal								
	Chulaimbo (R)			Kisumu (U)			Msambweni (R)			Ukunda (U)			Total no. of container visits (+ve)	Total no. of larvae and pupae	
	No. of container visits (+ve)	No. of larvae	No. of pupae	No. of container visits (+ve)	No. of larvae	No. of pupae	No. of container visits (+ve)	No. of larvae	No. of pupae	No. of container visits (+ve)	No. of larvae	No. of pupae		Larvae	Pupae
Jerrycan	458 (5)	19	4	290 (5)	11	2	164 (10)	155	16	368 (9)	100	18	1280 (29)	285	40
Bucket	738 (2)	20	9	727 (5)	26	4	766 (80)	1523	468	559 (19)	545	486	2790 (106)	2114	967
Pot	867 (346)	1605	301	527 (71)	171	39	20 (5)	236	32	21 (8)	482	33	1435 (430)	2494	405
Drum	99 (3)	25	5	270 (51)	263	48	113 (38)	2398	714	137 (35)	728	104	619 (122)	3414	871
SDC	742 (20)	151	22	681 (88)	284	39	145 (18)	547	134	178 (7)	191	36	1746 (133)	1173	231
AFC	66 (6)	50	19	79 (10)	21	8	33 (3)	12	5	53 (11)	891	99	231 (30)	974	131
Tire	33 (5)	40	3	243 (120)	607	182	18 (6)	541	127	64 (28)	1365	206	358 (159)	2553	518
Tree hole	32 (17)	153	49	4 (4)	35	8	0	0	0	0	0	0	36 (21)	188	57
Well	28 (5)	48	5	33 (0)	0	0	0	0	0	0	0	0	61 (5)	48	5
Other	960 (0)	0	0	747 (6)	39	2	81 (5)	163	24	116 (11)	175	49	1904 (22)	377	75

*Abbreviations*: *AFC* animal feeding containers, *SDC* small domestic containers, *+ve* positive

**Table 2:** Container types with larvae and pupae for indoor locations in western [Chulaimbo: Rural (R) and Kisumu: Urban (U)] sites and coastal [Msambweni (R) and Ukunda (U)] sites, Kenya

Container type	Western						Coastal								
	Chulaimbo (R)			Kisumu (U)			Msambweni (R)			Ukunda (U)			Total no. of container visits (+ve)	Total no. of larvae and pupae	
	No. of container visits (+ve)	No. of larvae	No. of pupae	No. of container visits (+ve)	No. of larvae	No. of pupae	No. of container visits (+ve)	No. of larvae	No. of pupae	No. of container visits (+ve)	No. of larvae	No. of pupae		Larvae	Pupae
Jerrycan	1729 (4)	3	0	2111 (6)	2	0	400 (1)	0	1	785 (5)	18	1	5025 (16)	23	2
Bucket	836 (0)	0	0	1108 (2)	2	0	1159 (9)	73	26	666 (5)	19	0	3769 (16)	94	26
Pot	317 (0)	0	0	204 (0)	0	0	58 (0)	0	0	27 (2)	45	45	606 (2)	45	45
Drum	186 (2)	5	0	246 (0)	0	0	201 (7)	195	91	294 (12)	147	28	927(21)	347	119
SDC	124 (0)	0	0	105 (0)	0	0	15 (1)	0	1	85 (1)	11	0	329 (2)	11	1
AFC	0	0	0	0	0	0	0	0	0	2	0	0	2 (0)	0	0
Tire	0	0	0	0	0	0	0	0	0	0	0	0	0	0	0
Tree hole	0	0	0	0	0	0	0	0	0	0	0	0	0	0	0
Well	0	0	0	0	0	0	0	0	0	0	0	0	0	0	0
Other	360 (1)	2	0	628 (0)	0	0	26 (0)	0	0	12 (0)	0	0	1026 (1)	2	0

*Abbreviations*: *AFC* animal feeding containers, *SDC* small domestic containers, *+ve* positive

### Container productivity

A total of 26,197 immatures were collected from the four study sites. Of these, 67.3% were identified as *Ae*. *aegypti.* We identified all the *Ae*. *aegypti* in this study as *Ae*. *aegypti aegypti* and none as *Aedes aegypti formosus*. Other mosquito species included: *Culex* spp. (30.2%), *Ae*. *simpsoni* (1.1%), *An*. *gambiae* (0.2%) and *Toxorhynchites* (1.1%). A total of 17,635 immature *Ae*. *aegypti* were collected from 1115 positive container visits in western and coastal regions. Buckets, drums, tires, and pots produced 77.4% (2704/3493) of *Ae*.*aegypti* pupae in the western and coastal study sites combined (Tables 1, 2). Drums, buckets, and pots, were the key *Ae*. *aegypti* containers indoors, producing nearly all (190/193) collected *Ae*. *aegypti* pupae (Table 2). Important outdoor containers in the coastal region were buckets, drums, and tires. While in the western region, pots and tires were the main producers (Table 1).

When productivity among regions was compared, the coastal region produced significantly more *Ae*. *aegypti* immatures (Kruskal-Wallis test, *χ* ^2^ = 77.3, *df* = 1, *P* < 0.0001) than the western region. In addition, productivity varied significantly between sites (*χ* ^2^ = 77.3, *df* = 3, *P* < 0.0001). Over 70% of the containers inspected in both western and coast regions were for water storage (drums, pots, buckets and jerrycans) (Table 3). They contributed 63.1 and 75.2% of larvae and pupae, respectively, in the coastal region, while in the western region they contributed 60.1 and 55.0% of larvae and pupae, respectively. Other container types, such as tires, small domestic containers, animal food troughs, tree holes, and wells produced the rest of the larvae and pupae collected. Of these, tires contributed most of the larvae and pupae. Water storage containers are important producers especially indoors where they contributed nearly all the larvae and pupae (Table 2). Analysis of relative pupal productivity by different container categories showed that buckets, drums and tires were the most productive in the coastal region overall with 35.6, 34.1 and 12.1% pupae per container type, respectively (Table 4). While in the western region pots and tires were the two most productive container types with 45.4 and 24.7% pupae per container, respectively (Table 5). Pupal index was notably higher (0.92) in the rural site of western Kenya than in the other three study sites (Ukunda: 0.47; Kisumu: 0.35; and Msambweni: 0.35).

**Table 3:** Productivity of water storage containers in comparison to other container types in rural (R) and urban (U) sites of western and coastal regions, Kenya

Container type	Western region						Coastal region					
	Chulaimbo (R)			Kisumu (U)			Msambweni (R)			Ukunda (U)		
	No. of container visits (+ve)	No. of larvae	No. of pupae	No. of container visits (+ve)	No. of larvae	No. of pupae	No. of container visits (+ve)	No. of larvae	No. of pupae	Container visit (+ve)	No. of larvae	No. of pupae
Jerrycan	2187 (9)	22	4	240 (11)	13	2	5,46 (11)	155	17	1153 (14)	118	19
Bucket	1574 (2)	20	9	1835 (7)	28	4	1925 (89)	1596	494	1225 (24)	564	486
Pot	1184 (346)	1605	301	731 (71)	171	39	78 (5)	236	32	48 (10)	527	78
Drum	285 (5)	30	5	516 (51)	263	48	314 (40)	2593	805	431 (47)	875	132
Total	5230 (362)	1677	319	3322 (140)	475	93	2863 (145)	4580	1348	2857 (95)	2084	715
Other container types												
SDC	866 (20)	151	22	786 (88)	284	39	160 (19)	547	135	263 (8)	202	36
AFC	66 (6)	50	19	79 (10)	21	8	33 (3)	12	5	55 (11)	891	99
Tire	33 (5)	40	3	243 (120)	607	182	18 (6)	541	127	64 (28)	1365	206
Treehole	32 (17)	153	49	4 (4)	35	8	0	0	0	0	0	0
Well	28 (5)	48	5	33 (0)	0	0	0	0	o	0	0	0
Other	1320 (1)	2	0	1375 (6)	39	2	107 (5)	163	24	128 (11)	175	49
Total	2345 (54)	444	98	2520 (228)	986	239	318 (33)	1263	291	510 (58)	2633	390

*Abbreviations*: AFC, animal feeding containers; SDC, small domestic containers; +ve, positive

**Table 4:** Relative importance of container categories in the coastal region, Kenya

Container category	No. of container visits (+ve)	Total no. of larvae in each container category	Proportion of larvae in each container category (%)	No. of containers with pupae	Total no. of pupae in each container category	No. of pupae per container type (Mean ± SD)	Frequency of containers with pupae (%)	Proportion of pupae in each container category (%)
Bucket	3150 (113)	2160	20.73	31	980	0.31 ± 6.43	0.98	35.64
Drum	745 (87)	3468	33.28	41	937	1.26 ± 11.70	1.30	34.07
Tires	82 (34)	1906	18.29	19	333	4.06 ± 11.51	0.60	12.11
SDC	423 (27)	749	7.19	13	171	0.40 ± 3.89	0.41	6.22
Pot	126 (15)	763	7.32	8	110	0.87 ± 4.17	0.25	4.00
AFC	88 (14)	763	7.32	5	110	1.18 ± 5.53	0.16	4.00
“Other”	235 (16)	338	3.24	7	73	0.31 ± 2.42	0.22	2.65
Jerrycan	1717 (25)	273	2.62	9	36	0.02 ± 0.35	0.29	1.31
Well	0	0	0	0	0	0	0	0
Treehole	0	0	0	0	0	0	0	0
Total	6566 (330)	10,420	100	133	2750			100

*Abbreviations*: *AFC* animal feeding containers, *SDC* small domestic containers, *+ve* positive

**Table 5:** Relative importance of container categories in the western region, Kenya

Container category	No. of container visits (+ve)	Total no. of larvae in each container category	Proportion of larvae in each container category (%)	No. of containers with pupae	Total no. of pupae	No. of pupae per container type (Mean ± SD)	Frequency of containers with pupae (%)	Proportion of pupa in each container category (%)
Pot	1915 (417)	1776	49.58	55	340	0.18 ± 1.22	2.87	45.39
Tire	276 (125)	647	18.06	41	185	0.67 ± 1.81	14.86	24.70
SDC	1652 (108)	435	12.14	12	61	0.04 ± 0.52	0.73	8.14
Drum	801 (56)	293	8.18	14	53	0.66 ± 0.66	1.75	7.08
AFC	145 (16)	71	1.98	6	27	0.19 ± 0.94	4.14	3.60
Bucket	3409 (9)	48	1.34	3	13	0.003 ± 0.15	0.09	1.74
Jerrycan	4588 (20)	35	0.98	3	6	0.001 ± 0.06	0.07	0.80
Well	61 (5)	48	1.34	2	5	0.08 ± 0.46	3.28	0.67
Other	2695 (7)	41	1.14	1	2	0.0001 ± 0.04	0.04	0.27
Treehole	36 (21)	188	5.25	13	57	1.58 ± 2.48	36.11	7.61
Total	15,578	3582	100.00	150	749			100

*Abbreviations*: *AFC* animal feeding containers, *SDC* small domestic containers, *+ve* positive

### Seasonal distribution of *Ae*. *aegypti* in wet containers

**Fig. 3:**
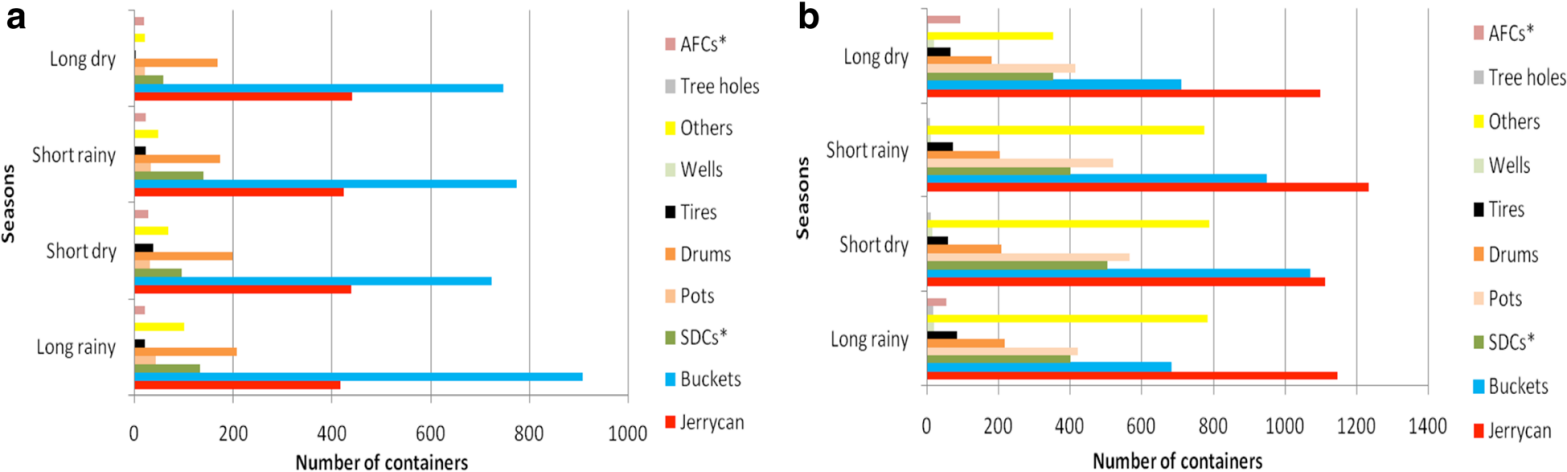
Seasonal abundance of *Ae. aegypti* larval habitats in **a** coastal [Msambweni (rural) and Ukunda (urban)] and **b** western [Chulaimbo (rural) and Kisumu (urban)] regions, between May 2014 and February 2016. Seasons: long rainy (April-June), short dry (July-September), short rainy (October-December); long dry (January-March). *Abbreviations*: AFC, animal feeding troughs; SDC, small domestic containers

**Fig. 4:**
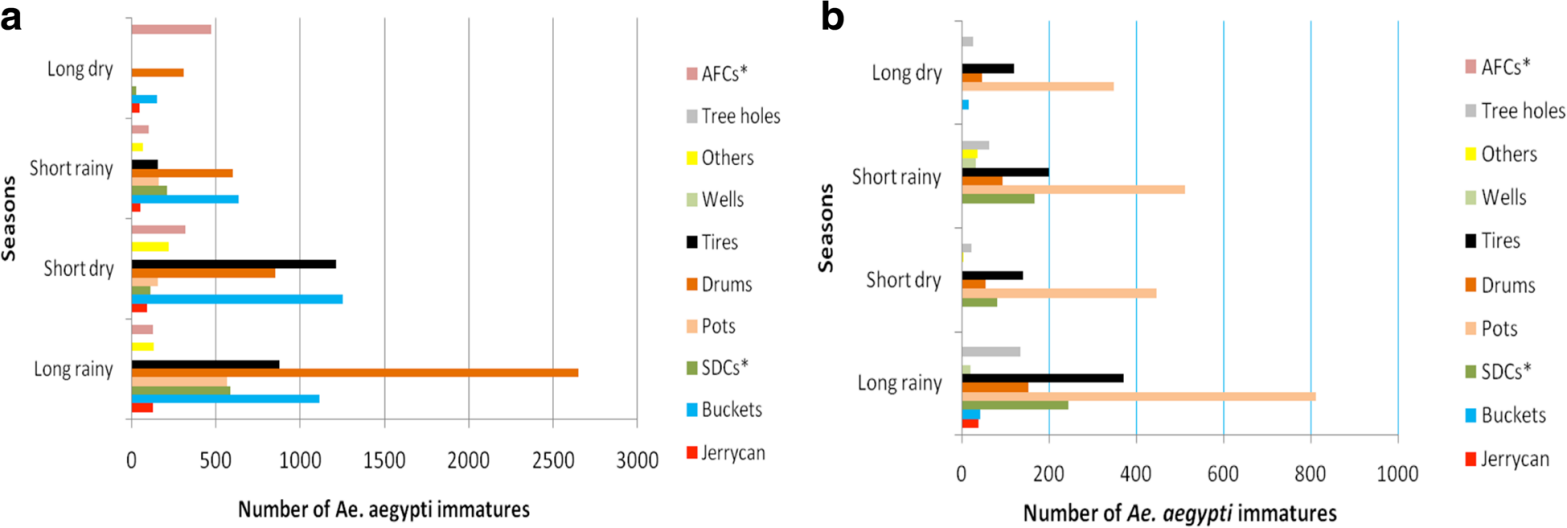
Seasonal abundance of *Ae. aegypti* immatures in different container types in **a** the coastal (Msambweni and Ukunda) and **b** western (Chulaimbo and Kisumu) regions, between May 2014 to Feb 2016. Seasons: long rainy (April-June), short dry (July-September), short rainy (October-December); long dry (January-March). *Abbreviations*: AFC, animal feeding troughs; SDC, small domestic containers

The abundance and distribution of different types of containers followed a consistent pattern over the dry and rainy seasons in western and coast regions (Fig. 3). Immature *Ae*. *aegypti* were found in more container types during the three seasons, i.e. long rainy (April-June), short dry (July-September), and short rainy (October-December), than in the long dry (January-March) season in both regions (Fig. 4). In the coast region, during the long rainy season except for tree holes, wells and others, nearly all container types were important producers of *Ae*. *aegypti* immatures. In contrast, in the western region pots were the main producers of *Ae*. *aegypti* immatures in all seasons (Fig. 4b). Productivity among seasons varied significantly (Kruskal-Wallis test, *χ* ^2^ = 83.2, *df* = 3, *P* < 0.0001) with more immatures in long rainy seasons and the least in long dry seasons.

### Oviposition activity

A total of 59,641 eggs were collected from 3538 ovitraps during the 24-month period in western (Chulaimbo and Kisumu) and coastal regions (Msambweni and Ukunda). Of these, 97.1% were of *Ae*. *aegypti*, 2.3% *Ae*. *simpsoni* and 0.5% *Culex* spp. Significantly more ovitraps were positive for *Ae*. *aegypti* eggs outdoors than indoors (*χ* ^2^ = 584.3, *df* = 1, *P* < 0.0001) and contained more eggs both in the western region [436 (46. 8%) containing 24,949 eggs vs 37 (4.0%) containing 920 eggs] and the coastal region [418 (50.0%) containing 26,618 eggs vs 160 (19.1%) containing 7154 eggs]. Mean number of eggs per trap (Mean egg index) was significantly higher (Kruskal-Wallis test, *χ* ^2^ = 91.2, *df* = 3, *P* < 0.0001) in the three seasons, i.e. long rainy (April-June), short dry (July-September) and short rainy (October-December) than in the dry season (January-March) for both regions. However, the percentage of positive ovitraps (Ovitrap positivity index) and mean egg index were notably higher (*P* < 0.0001) in the coastal region.

## D**iscussion**

In this study, we report a notable preference for outdoor breeding by *Ae*. *aegypti,* contrary to earlier findings from the region [13], but consistent with other recent findings elsewhere [20, 25, 41–43]. This is clearly indicated by significantly more *Ae*. *aegypti* immatures found in containers located outdoors, despite a higher number of containers with water indoors (Tables 1, 2). Further in support of this finding, oviposition surveys in all the study sites show significantly higher oviposition activity by *Ae*. *aegypti* outdoors than indoors. Drums, buckets, discarded tires, and small domestic containers are the key containers for *Ae*. *aegypti* development in all study sites, consistent with other studies [2, 19, 22, 44, 45]. In addition, water storage containers produced most of the immatures recorded, underscoring the importance of such category of containers in these regions. Significantly higher *Ae*. *aegypti* breeding activity was observed in the coastal region than in the west, even though the west had a notably higher number of potential breeding habitats. Container habitats of *Ae*. *aegypti* are consistently available throughout the year in all the study sites; however, *Ae*. *aegypti* was found to breed in more container types during the wet and short dry seasons.

The observed increased outdoor breeding activity by *Ae*. *aegypti* suggests an adaptation to outdoor and peridomestic habitats [20], a trend that is most likely to have epidemiologically important implications for vector control practices and prevention of virus transmission. This calls for more emphasis to be placed on the outdoor larval habitats in targeted source reduction measures in the study areas. Widespread use of insecticide impregnated bed nets in our study sites [33, 46, 47] could be a factor accounting for the preference of outdoor breeding by *Ae*. *aegypti*. Traditionally *Ae*. *aegypti* has been demonstrated to be a domestic species that is strongly endophilic occurring in bedrooms, kitchens, bathrooms and living rooms, where it rests on walls, hanging cloths, bednets and under furniture [48–50]. This may bring resting females into contact with insecticide-treated materials that are likely to kill or at least repel them from indoor resting sites. In our ongoing study (unpublished data), *Aedes* mosquitoes have been found to be susceptible to insecticides that are commonly used in bednets. However, further studies are required to confirm and possibly establish factors that can lead to this observed trend toward outdoor and peridomestic habitats.

Low indoor productivity in our study sites can also be attributed to human activities related to the use of domestic water receptacles. Most indoor containers are commonly used for hygiene, cooking and drinking and are subject to frequent emptying and cleaning which can effectively interrupt mosquito development. They are therefore much less likely to harbor *Ae*. *aegypti* immatures [22, 25]. Moreover, most of the indoor containers for water storage were often covered; this could have possibly contributed to many of them being unproductive. Containers with covers have been found to have a lower probability of infestation by *Aedes* mosquitoes [22] by preventing gravid females from accessing oviposition sites [45, 51].

Tires provided good breeding sites for *Aedes* mosquitoes and are responsible for producing > 30% of immatures collected from all larval habitats in outdoor locations of urban areas. The importance of tires as breeding sites of *Ae*. *aegypti* has been highlighted before [18, 19, 51–53], and recycling as a means to manage used tires in dengue control has gained popularity around the globe [30]. In the urban areas of our study sites, recycling of tires is limited to small-scale use for making sandals and soles of shoes. Therefore, intensifying other recycling options [30] is highly recommended. In addition, storage of tires in a dry environment and proper disposal of used tires should be encouraged in instances where recycling may not be feasible.

Buckets and jerrycans were found in large numbers in all the study sites, but their importance as breeding sites for *Ae*. *aegypti* mosquitoes was limited to the coastal region, especially outdoors. Unlike buckets, many jerrycans were consistently present indoors in the study sites, but they were not equally productive. Some studies have identified jerrycans as among the preferred outdoor breeding habitats [18, 19], contrary to the situation in our study sites. Low productivity by this container type can possibly be attributed to their popular usage in short-term storage of water and therefore being subject to frequent emptying and cleaning, which effectively interrupt the breeding cycle of *Ae*. *aegypti* immatures [30, 54]. Water-holding containers that are in frequent use within the domestic environment were observed to be less likely to harbor *Aedes* immatures [22], and this can make water storage possible without necessarily creating breeding sites for *Aedes* mosquitoes.

Animal feeding containers were only found in the coastal region, and their importance was particularly noticed in urban site where concrete troughs and cut tires, popular as watering points for cattle and goats in backyards of some homesteads, provided good larval sites for *Ae*. *aegypti* mosquitoes. The importance of water storage containers is due to the fact that they can hold sufficiently large volumes of water for considerably longer periods that are adequate for complete larval development. However, breeding in these containers can be eliminated by provision of tight fitting covers and mesh screens to prevent access by gravid mosquitoes [30, 45, 51].

In this study, removal or proper management of key containers could result in over a 65% reduction in *Ae*. *aegypti* immature population. This would translate into a corresponding reduction in *Ae*. *aegypti* population in the study areas, since pupae can be used as proxy estimates of adult *Ae. aegypti* mosquitoes [2].

Higher *Ae*. *aegypti* breeding activity at our coastal study sites can be attributed to many factors. Our coastal urban site is characterized by unplanned settlements and a large population of low income earners in the informal business sector, where poor hygiene coupled with inadequate water, sewer and waste management systems are common. These conditions have been found to contribute significantly to the proliferation of breeding sites for *Ae*. *aegypti* [55, 56]. In addition the practice of keeping domestic animals in backyards of these neighborhoods, probably to boost the low income levels, provides more breeding opportunities for the highly adaptive *Ae. aegypti* mosquito [57] which readily exploits the water receptacles meant for animal drinking and feeding. The comparatively higher breeding activity by *Ae*. *aegypti* observed in the coastal sites could possibly account for the dengue outbreaks reported mostly in the coastal region as observed in the recent past [9].

The year-round activity of *Ae*. *aegypti* can be associated with the lack of a reliable water supply system, and reliance on borehole water, well and rain water collection, necessitating water storage in households. During the long dry season, in particular, drums, buckets and pots become important producers of *Ae*. *aegypti* immatures in both regions. *Ae*. *aegypti* breeding in our study sites is closely related to the rainfall patterns in the two regions, with higher production of immatures during the rainy season, when environmental conditions are also optimal for adult activity, which translates into higher productivity of containers.

Of the high number of water-holding containers inspected, only a few were productive for *Ae*. *aegypti*, and most of them were located outdoors. Identification of key larval habitats for *Ae*. *aegypti* as part of vector control programs [45, 58] will help target larval source reduction measures. Dengue is primarily a problem of human activities that create breeding opportunities for potential vectors, the control of which can be achieved by physical means [30, 50]. In our study, good management practices for water storage containers [22, 50], recycling and proper disposal of discarded tires and small domestic containers [30] are recommended in order to achieve a significant reduction in *Aedes* population outdoors, especially during the rainy season when the latter type of containers become important breeding habitats. In addition, provision of reliable piped water supply to households in the study sites throughout the year would reduce storage of water in containers and thus control *Ae*. *aegypti* development sites.

The results of this study should be interpreted with caution since we systematically and repeatedly sampled households that were randomly selected for larval and ovitrap surveys. Though this sampling design enabled us to conduct the surveys over an extended period, we note that the study population may change its behavior over time, which in turn, may impact our findings. In addition, although our sample size of 20 sentinel houses in each of the four study sites may have been statistically inadequate, we were not able to inspect other larval sites beyond the large number in the individual sentinel households, given limited resources and larval surveys being labor intensive.

We distinguished *Ae*. *aegypti* subspecies morphologically following keys as provided by Edwards [37], Mattingly [38] and Huang [39]. While morphological identification of the subspecies is not optimal, the more sensitive molecular techniques are yet to be fully developed [59], and are not readily available in field conditions. *Ae*. *aegypti aegypti* has been reported to be predominantly domestic and peridomestic, while *Ae*. *aegypti formosus* mostly sylvatic [57, 59–61] . We identified all the *Ae*. *aegypti* in this study as *Ae*. *aegypti aegypti*. Given that all our samples are from the domestic or the immediate peridomestic environment, (within a range 10 m around sampling houses), we are confident that the presence of *Ae. aegypti formosus* did not have a significant impact on our findings.

## Conclusions

The results of this study indicate that *Ae*. *aegypti* breeding habitats are abundant outdoors and are diverse both in the coastal and western regions of Kenya. However, a limited number of container types are responsible for the majority of the adult vector production. Targeting source reduction efforts toward these productive container types may be a cost-effective way to reduce the abundance of the dengue vector and arboviral transmission in these regions.

## Abbreviations

CDC: Centers for Disease Control
DENV: Dengue virus
KEMRI: Kenya Medical Research Institute
PAHO: Pan American Health Organization
VBDCU: Vector borne disease control unit
WHO: World Health Organization
